# Expert curation for building network-based dynamical models: a case study on atherosclerotic plaque formation

**DOI:** 10.1093/database/bay031

**Published:** 2018-04-04

**Authors:** Amel Bekkar, Anne Estreicher, Anne Niknejad, Cristina Casals-Casas, Alan Bridge, Ioannis Xenarios, Julien Dorier, Isaac Crespo

**Affiliations:** 1 Vital-IT group, SIB Swiss Institute of Bioinformatics, Quartier Sorge, Bâtiment Génopode, 1015 Lausanne, Switzerland; 2 Swiss-Prot group, SIB Swiss Institute of Bioinformatics, 1 Michel Servet, 1211 Geneva 4, Switzerland

## Abstract

Knowledgebases play an increasingly important role in scientific research, where the expert curation of biological knowledge in forms that are amenable to computational analysis (using ontologies for example)–provides a significant added value and enables new types of computational analyses for high throughput datasets. In this work, we demonstrate how expert curation can also play a more direct role in research, by supporting the use of network-based dynamical models to study a specific biological process. This curation effort is focused on the regulatory interactions between biological entities, such as genes or proteins and compounds, which may interact with each other in a complex manner, including regulatory complexes and conditional dependencies between co-regulators. This critical information has to be captured and encoded in a computable manner, which is currently far beyond the current capabilities of automatically constructed network. As a case study, we report here the prior knowledge network constructed by the sysVASC consortium to model the biological events leading to the formation of atherosclerotic plaques, during the onset of cardiovascular disease and discuss some specific examples to illustrate the main pitfalls and added value provided by the expert curation during this endeavor.

**Database URL**: http://biomodels.caltech.edu

## Introduction

New types of computational analyses, such as network-based dynamical models, take advantage of the enormous amounts of data that biologists today are able to generate at relatively low cost. However, expert curation remains critical to transform available data—including publications—into computable knowledge. Network-based dynamical models are abstractions of reality that can be used to simulate the behavior of complex biological systems and to predict their responses to environmental stimuli and disease, providing an essential support for hypothesis generation and experimental design (1, 2). In this work, we focus on network-based dynamical models in the Boolean framework (3–6), where states of the constituent elements, the nodes of the network, are Boolean variables that can take only two values: 0 (inactive) and 1 (active). Interactions between nodes are specified using logical rules consisting of a combination of AND, NOT and OR logical operators.

Network-based dynamical models can be directly inferred from experimental data, such as gene expression data, molecular concentrations or phenotypes, in normal and perturbed states (7, 8). The parameters of the model are obtained by fitting the model to the experimental data. Alternatively, network-based dynamical models can be created using prior knowledge obtained from the literature––resulting in a list of regulatory interactions termed a prior knowledge network (PKN; 9, 10). This approach requires expert curation of regulatory interactions, which can be performed specifically on the model, or which can be sourced from existing curated knowledgebases. Expert curators summarize experimental findings and encode the information in such a way that it can be easily queried and linked to other types of data by using shared identifiers and ontologies (11). Examples of such curated knowledgebases are UniProtKB/Swiss-Prot (12) and IMEx (13). The two approaches to construct network-based dynamical models (inferred from data and derived from prior-knowledge) can also be combined (14–16). Indeed, it has been demonstrated that incorporating pre-existing biological knowledge improved inferring causal molecular networks from data (17). However, human expertise remains essential for the construction of useful PKNs (18–20).

In this work, we describe the creation of a PKN, which describes regulatory interactions that play a role in the formation of atherosclerotic plaques (Supplementary Table S1). This PKN can be used as the basis for the construction of a network-based dynamical model describing the formation of atherosclerotic plaques during the onset of cardiovascular disease. The PKN includes logical rules that describe complex interactions between multiple network components––rules which cannot be encoded automatically, from data––and in the second part of this work, we illustrate how these logical rules improve the quality of the network-based dynamical models derived from the PKN.

## Constructing a PKN of the formation of atherosclerotic plaques

### Motivation for building a PKN encoding regulatory logic rules

**Figure 1.**
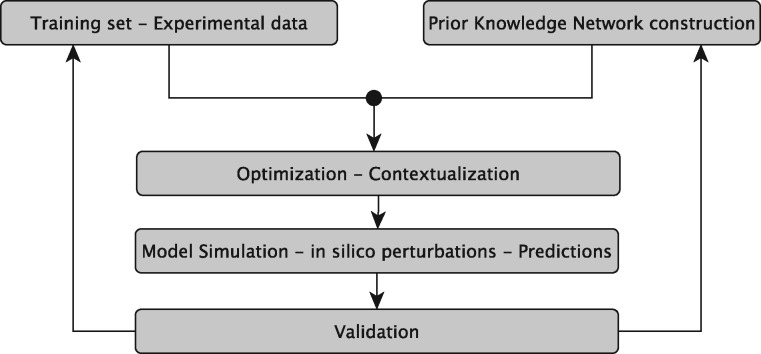
The sysVASC dynamical model of atherosclerotic plaque formation flow chart. The sysVASC consortium attempts to elucidate pathological mechanisms involved in the onset and progression of cardivascular disease and to identify and validate novel biology-driven key molecular targets for therapeutic intervention by *in silico* simulation of the main driving biological processes. Among those processes, the formation of atherosclerotic plaque plays a central role. In this work, we use *in silico* simulation of a dynamical model based on an expert curated PKN, to optimize the model and to generate testable predictions for further validation. Such predictions are used to iteratively improve the model.

The sysVASC consortium (Systems Biology to Identify Molecular Targets for Vascular Disease) consists of 17 European partners funded by the European Union’s Seventh Framework Program for research, technological development and demonstration (http://www.sysvasc.eu/). The consortium aims to elucidate the pathological mechanisms underlying the onset and progression of cardiovascular disease and to identify and validate novel biology-driven key molecular targets for therapeutic intervention. As a part of a comprehensive systems medicine approach developed for this purpose, the consortium developed the platform for *in silico* simulations of cardiovascular disease-related biological processes, which ultimately relies on the construction of network-based dynamical models (Figure 1). Network-based dynamical models can describe the evolution of a biological system in time, and provide the means to investigate the effect of perturbations on that evolution. They can be used to predict key molecular events in normal and pathological states, serving as a guide for experimental design and hypothesis generation (7). These aims fit very well with the goals and expectations of many experimental research projects including those of the sysVASC consortium.

One of the main limitations we encounter when attempting to describe the overall dynamics of a complex system is that the related kinetic parameters are mostly unknown, or may have to be learned from a significant amount of (potentially costly) experimental data. One way to deal with this problem is to adopt a modeling formalism that avoids the need for explicit determination of kinetic parameters (21, 22). Such formalisms include logic-based models, such as those based on Boolean logic, which adopt a simplified description of a given biological system (3, 23). In Boolean models, the state of the constituent elements can be either ‘active’ or ‘inactive’, and the evolution of these states is defined by the state of their direct regulators according to specific logical rules. The entire collection of these rules, encoded as regulatory logic functions, comprise a Boolean model. Although these models lack temporal units, they can be used to explore properties of biological systems such as the presence of attractors (states of convergence, often phenotypes such as healthy or disease states) and the alterations or perturbations that drive transitions between them (24).

## Description of the methodology used to build the PKN: expert curation by Swiss-Prot team

Expert biocuration of the PKN was performed by the Swiss-Prot curation team, which consists of experienced (generally PhD-level) biologists or biochemists with a strong experience in wet lab research.

Expert curators assimilate information from a number of sources, including full text publications, analyse and reconcile conflicting results and integrate the data into a concise but comprehensive network of functional interactions. In order to ensure consistency, we used controlled vocabularies and ontologies for all terms: gene names come from the official HGNC nomenclature (https://www.genenames.org/), chemical compounds from ChEBI (https://www.ebi.ac.uk/chebi/), tissues or cells from Uberon (http://uberon.github.io/) and biological processes or complexes from Gene Ontology (http://www.geneontology.org/).

The PKN is composed of: (i) nodes representing biological entities; (ii) regulatory interactions between biological entities (edges) and (iii) logical rules that describe the interplay between (co-)regulators. Nodes representing biological entities in the network (1) may describe proteins, complexes, small molecules, biological processes, or phenotypes. Regulatory interactions (edges; 2) between source and target nodes may positively or negatively regulate the activity of the latter. These simple regulatory interactions reduce biological processes as diverse as the regulation of transcription, translation, transport and post-translational modification to simple activation or inhibition. Logical rules (3) that specify how co-regulators interact with respect to a target node are encoded using three basic logic operators: ‘OR’, ‘AND’ and ‘NOT’.

## Description of the PKN

Atherosclerotic plaque formation is a very complex process that initiates with lipid accumulation followed by monocyte infiltration, platelets recruitment and lipid core formation leading a chronic systemic inflammation state. In this process, many pathways and crosstalks between different cell types and tissues are involved. To build the PKN, Swiss-Prot curation team mainly focuses, to start, on the inflammasome activation produced by cholesterol crystals in macrophages during chronic systemic inflammation, which induces interleukine-1 beta (IL1B) production (25, 26). Starting from this process, they extended the curation to the upstream mechanisms that lead to the inflammasome activation, but they also annotated the downstream consequences that end in the atherosclerotic plaque formation.

The PKN is composed of a total of 729 nodes, of which 432 are proteins and 297 are other entities such as metabolites or biological processes (Supplementary Table S1). These nodes are linked by 3406 interactions (rows in Supplementary Table S1) that describe the effect of one or more regulatory nodes (column B) on their corresponding target nodes (column E); 2878 of these edges describe activation (-> in column D) and 528 describe inhibition (-| in column D) of the target nodes. Among these 3406 interactions 1841 complex regulations are encoded with logical operators while 1565 are simple activatory or inhibitory edges of one node over another.

The diameter of the PKN i.e. the maximum eccentricity (the greatest number of edges between a node n and any other node) of any node is 14. Its radius -i.e. the minimum eccentricity of any node is 1. The PKN is also fully connected with one connected component meaning that there is always an undirected path between each pair of nodes. The shortest paths constitute 10% of the network. The average number of neighbors for nodes is 6. It has 8 self-loops.

## Added value of expert biocuration: illustrative examples

Although some efforts have been made to collect directed and signed cause-and-effect relationships from automated text mining (27), text-mining methods are only really accurate for named entity recognition (28). Expert curation remains the only accurate means to extract complex regulatory relationships from text with the degree of accuracy required to construct computational models. Usually these models include hundreds of interactions, and without the help of expert curation, high error rates in the identification of individual entities and their relations would make the resulting models unusable. Signal transduction and gene regulatory networks result from complex interactions and from the regulation of elements acting in concert rather than independently of each other. Expert curation allows us to capture this complexity and encode it in the form of logical rules.

To illustrate the importance of this expert curation in building PKNs and encoding complex regulations, we will focus on the regulation of three central components of the cardiovascular disease and atherosclerosis plaque formation network: peroxisome proliferator-activated receptor alpha (PPARA), MAPK1/3 and IL1B secretion. For each of these components, we will demonstrate how the correct capture and encoding of the dependencies between its co-regulators is essential to correctly predict its state in health and disease. For this purpose, we compare predictions made on network-based dynamical models extracted from the PKN to data extracted from the literature on the state of these model constituent nodes (genes) in healthy and disease phenotypes (Supplementary Table S2). Supplementary Table S2 was built by collecting information from the literature reporting the correlation between states of PKN nodes (column A) and cardiovascular disease (column B). For nodes positively correlated with cardiovascular disease, a state 0 was assigned to healthy phenotype (column F) and state 1 to diseased phenotype (column G). Similarly, for nodes negatively correlated with cardiovascular disease, states 1 and 0 were assigned to healthy and diseased phenotype, respectively.

For each component (PPARA, MAPK1/3 and IL1B secretion), we created two versions of a network-based dynamical model from the PKN keeping all the interactions between the component as target node and its regulators. The first version of the model retains the logical rules from the PKN, while the second version of the model was built by keeping only the information on the topology and sign of interactions, neglecting the prior knowledge concerning the specific regulatory logic rules and adopting an inhibitory dominant system instead, which is commonly used by default (29–32). In the latter case, a node is considered active if at least one of its activators (if any) and none of its inhibitors (if any) are active; the node is considered inactive otherwise.

For each model, we considered the healthy and disease phenotypes obtained from the literature separately. For each phenotype, we fixed the state of all input nodes to the corresponding value in Supplementary Table S2. We used boolSim (29) to predict the state of the output node and compare it with the expected state according to the phenotype obtained from literature.

## PPARA regulation

PPARA is a nuclear receptor activated by natural ligands such as fatty acids, and it’s activation seems to inhibit early processes contributing to atherosclerotic plaque formation (33). PPARA is activated by PARGC1A, acting singly or jointly with either TNF (10) SIRT1 (34) or in the absence of MAPK14 (35). These relations can be formulated with logical rules as follows:

- PPARA = (TNF AND PPARGC1A).
- PPARA = (TNF AND SIRT1).
- PPARA = (NOT MAPK14 AND PPARGC1A).

These rules were encoded in a model, which subsequently predicted the state of PPARA as active in healthy states and inactive during atherosclerotic plaque formation (true).

The second model, using only information on the topology and sign of interactions (i.e. combining activators and inhibitors with OR logic gate and using the inhibitory dominance rule), included the following rule:

- PPARA = (SIRT1 OR PPARGC1A OR TNF) AND NOT MAPK14.

**Figure 2.**
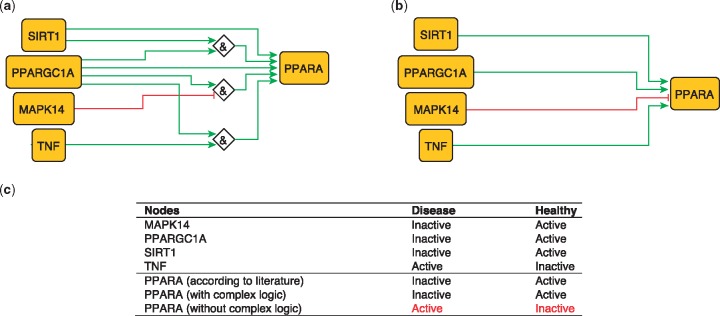
Network-based dynamical model for the action of regulators upon PPARA with (**a**) and without (**b**) using logic rules from the PKN. Green and red edges are activatory and inhibitory, respectively. Diamond nodes are ‘AND’ gates. (**c**) Input/output node states (either active or inactive) in disease and healthy phenotypes according to literature and model predictions. States that do not behave as expected from the literature are shown in red. Input nodes states are fixed according to information collected from literature (Supplementary Table S2).

This model predicts a constitutively active PPARA, which is contradictory to what has been observed (33; Figure 2). This illustrates the added value of expert curation in the description of complex signaling functions and their integration, a task that is beyond machines at the current time.

The model built using logic rules can be found in SBML-Qual format (36) in BioModels (2) with the identifier MODEL1712240001.

## MAPK1/3 regulation

MAPK1 and MAPK3 are serine/threonine kinases that play a central role in MAP kinase signal transduction pathway. They are essential constituents of IFNG-mediated activation of STAT1 as well as the expression of key genes implicated in atherosclerosis, and the uptake of modified lipoproteins by macrophages (37). Among MAPK1/3 regulators, Glyoxal (GO) and Methylglyoxal are metabolites of glycolysis and lipid peroxidation that are increased in patients with diabetes (38). They are highly reactive compounds that have been shown to severely inhibit PDGF-induced activation of MAPK (39).

This regulation of MAP kinases can be encoded as:

- MAPK1 = (NOT GO AND PDGFB) OR (NOT methylgyoxal AND PDGFB).
- MAPK3 = (NOT GO AND PDGFB) OR (NOT methylgyoxal AND PDGFB).

**Figure 3.**
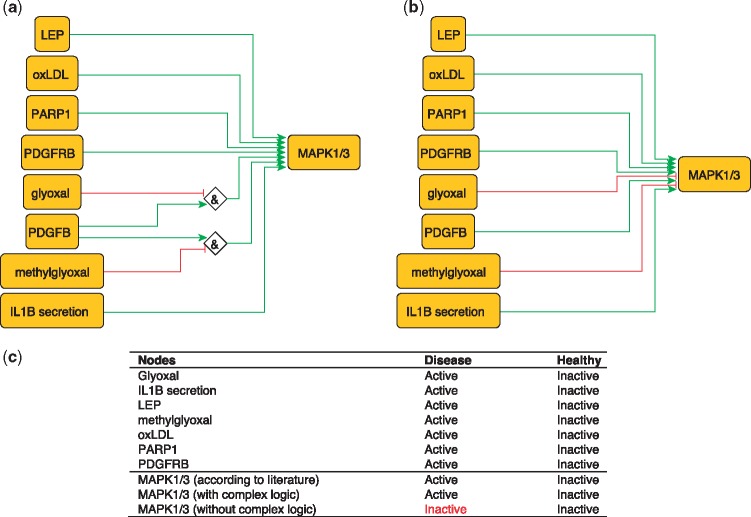
Network-based dynamical model for the action of regulators upon MAPK1/3 with (**a**) and without (**b**) using logic rules from the PKN. Green and red edges are activatory and inhibitory, respectively. Diamond nodes are ‘AND’ gates; (**c**) Input/output node states (either active or inactive) in disease and healthy phenotypes according to literature and model predictions. States that do not behave as expected from the literature are shown in red. Input nodes states fixed according to information collected from literature (Supplementary Table S2).

These rules are part of a model that correctly predicts MAPK1/3 states in the healthy and disease phenotypes (Figure 3). Failure to incorporate this information will lead to incorrectly predict the inactivation of MAPK1/3 during atherosclerotic plaque formation.

The model built using logic rules can be found in SBML-Qual format in BioModels with the identifier MODEL1712240003.

## IL1B secretion regulation

**Figure 4.**
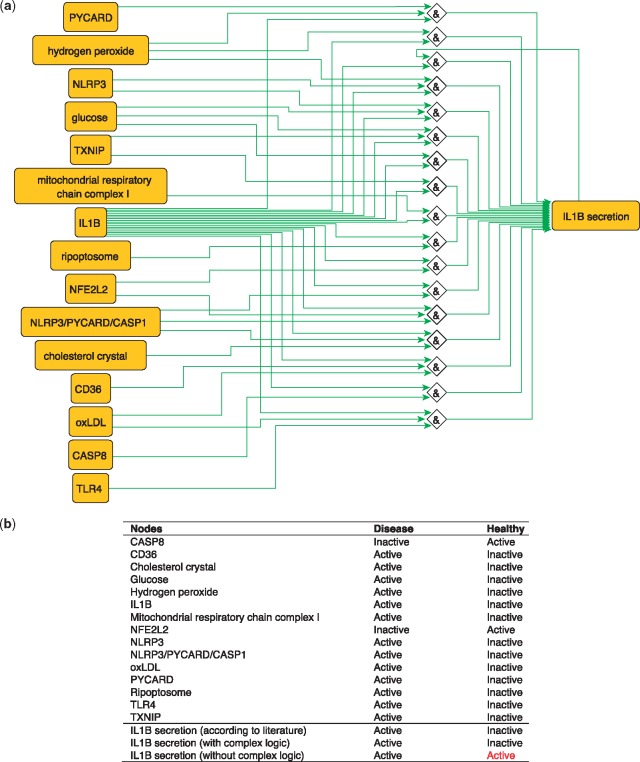
(**a**) Network-based dynamical model for the action of regulators upon IL1B secretion using logic rules from the PKN. Green edges are activatory edges. Red edges are inhibitory edges. Diamond nodes are ‘AND’ gates. (**b**) Input/output node states (either active or inactive) in disease and healthy phenotypes according to literature and model predictions. States that do not behave as expected from the literature are shown in red. Input nodes states are fixed according to information collected from literature (Supplementary Table S2).

IL1B is a proinflammatory cytokine. The production of IL1B occurs in two main steps, where inflammatory signals stimulate the synthesis of pro-IL1B, while inflammasome assembly leads to CASP1 activation and pro-IL1B processing to form the active cytokine IL1B which is then secreted (40). These steps are represented in the PKN by two distinct nodes: ‘IL1B’, representing the synthesis of the cytokine and ‘IL1B secretion’ that describes its release. IL1B secretion is subject to complex regulation so the proper integration of signals coming from its various regulators using logical functions is critical. Regulators of IL1B secretion are very diverse, ranging from metabolites such as glucose and hydrogen peroxide (41) to complexes such as the NLRP3/PYCARD/CASP1 inflammasome (42) and the mitochondrial respiratory chain complex I (17) and all must be correctly integrated according to experimental knowledge in order to produce a model that makes correct predictions about the secretion of IL1B in the healthy state (Figure 4). When this interplay is removed, and regulators are considered separately (i.e. combined with OR logic gates), then the network is not able to correctly predict the secretion of IL1B in the healthy state. The model built using logical rules can be found in SBML-Qual format in BioModels with the identifier MODEL1712240002.

## Other sources of model misbehavior: PKN contextualization

It is worth noting here that even when available information from the scientific literature is correctly encoded models may fail for other reasons––including gaps in the literature (unknown mechanisms), model oversimplification, or a failure to correctly contextualize a model for a given biological system. With respect to the latter, PKNs may include interactions described in different biological contexts and experimental conditions; these interactions could be absent or inactive in the specific biological process under study. Therefore, creating a dynamical model based on a PKN usually requires several rounds of optimization, or contextualization, where the model is trained against experimental data measured on the biological process of interest. The contextualization usually consists in pruning edges that are causing apparent dynamical model misbehavior, until the dynamical model is able to successfully reproduce the experimental data or point out a mismatch, which can be overcome with additional literature search or even dedicated experiments (guiding the experimental design). This optimization can be done manually, but for large PKNs this can become difficult and time consuming. Alternatively, several tools exist that can automatize this procedure (2, 14, 16, 43, 44).

**Figure 5.**
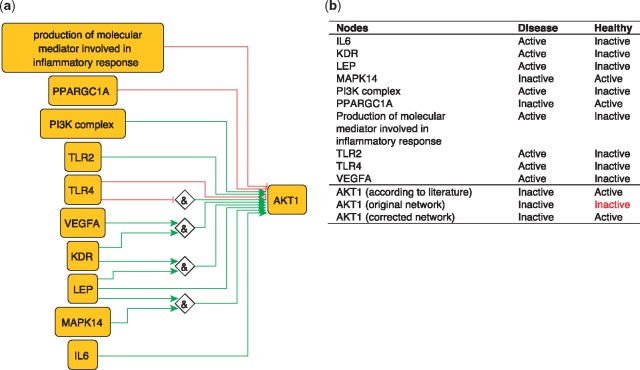
(**a**) Network-based dynamical model for the action of regulators upon AKT1 built using logic rules from the PKN. Green edges are activatory edges. Red edges are inhibitory edges. Diamond nodes are ‘AND’ gates. (**b**) Input/output node states in disease and healthy phenotypes according to literature and model predictions. The corrected model succeeds to reproduce the healthy and disease states of AKT1 while the original one reproduced correctly only the disease state. Input nodes states are fixed according to information collected from literature (Supplementary Table S2).

To illustrate the importance of this optimization, we use the example of AKT1 from our PKN. AKT1 has been shown to have a protective effect by inhibiting atherosclerosis (45). However, the network-based dynamical model extracted from our PKN, which includes all identified regulators of AKT1, nevertheless fails to reproduce the active state of AKT1 in the healthy phenotype. This is due to the inhibitory edge from PPARGC1A to AKT1––which represents the ability of PPARGC1A to reduce phospho-AKT1 (Ser-473) levels in skeletal muscle cells (46)––that prevents the activation of AKT1. Removing this edge is sufficient to rescue proper behavior of AKT1 (Figure 5), suggesting that this inhibitory effect should not be transposed to the atherosclerotic context.

## Conclusions

Although biologists today are able to generate enormous amounts of data at relatively low cost, expert curation remains critical to transform available data––including publications––into computable knowledge. The Swiss-Prot competence center in biocuration has a long tradition in the development, the annotation and reviewing of reference knowledgebases such as UniProtKB/Swiss-Prot (47). In this work, we highlight the critical added value that the application of expert curation can bring directly to research projects, in particular for the construction of PKNs intended to be used as network-based dynamical models for the study of biological processes, such as diseases.

There are two main reasons why network-based dynamical models have generated interest as a means to study complex biological systems. First, such systems usually comprise a huge number of elements; experimentally checking all constituent elements under all possible biological conditions would be very costly. Second, given the stochastic nature of biological systems, independent elements of the system have a limited utility either as disease biomarkers or as intervention points; more robust and reliable predictions and responses should be obtained from combinations of biomarkers and targets, respectively. Of course, the search space of combinations of elements in such a system is enormous, rendering a trial and error approach unfeasible. Simulations on network-based dynamical models can help to evaluate and prioritize the best disease biomarkers or therapeutic target candidates for further experimental evaluation, reducing the search space and guiding the experimental design.

We use our work on the onset and progression of cardiovascular disease as a means to illustrate the essential role for human intelligence–expert curation––to correctly identify and encode complex regulatory interactions from experimental literature. Failure to encode these relationships correctly will dramatically change the behavior of the model and the derived predictions. This logical modeling framework is particularly suited to model complex and poorly understood systems where continuous modeling is impossible due to the lack of quantitative information and/or the computational resources that quantitative approaches require. The models we have produced here are available for others to use as a basis to create network-based dynamical models for atherosclerotic plaque formation process or specific pathways involved in cardiovascular disease progression.

Based on our experience in this field, we strongly recommend the incorporation of (and budgeting for) expert curation as an essential element in any computational modeling project from the outset. We also recommend that network-based dynamical models should be fine-tuned by contextualization to the specific biological system under study. Computational modeling requires a wide range of expertise and provides a fantastic opportunity to catalyze interdisciplinary research involving experts as diverse as biocurators, experimental biologists and modelers.

## Supplementary data

Supplementary data are available at *Database* Online.

## Funding

This project has received funding from the European Union’s Seventh Framework Program for research, technological development and demonstration under grant agreement no: 603288.

*Conflict of interest*. None declared.

## Supplementary Material

Supplementary Data
